# Widespread prevalence of CD19 exon 5–6 skipping in primary pediatric B-Cell acute lymphoblastic leukemia patients

**DOI:** 10.1186/s40348-025-00207-y

**Published:** 2025-11-17

**Authors:** Devesh Srivastava, Anurag Gupta, Nishant Verma, Ashish Misra

**Affiliations:** 1 Department of Biotechnology, Indian Institute of Technology Hyderabad, Kandi, Sangareddy 502285 India; 2 King George Medical University, Lucknow, 226003 India

**Keywords:** B-ALL, CAR-T therapy, CD19, Aberrant splicing, RNA-binding proteins

## Abstract

**Background:**

B-cell acute lymphoblastic leukemia (B-ALL) is characterized by the malignant burgeoning of abnormal B-cell lymphoblasts. In recent years, the use of chimeric antigen receptor T-cell (CAR-T) therapy which targets CD19 antigen present on the surface of B-cells, has gained significant attention as a treatment option against aggressive and refractory forms of B-ALL. However, the loss of CD19 antigen on B-cell surface due to aberrant splicing under therapy pressure has been suggested as one of the main factors for the emerging CAR-T therapy resistance. The primary aim of this study was to elucidate the presence and characteristics of aberrant CD19 splicing patterns in pediatric B-ALL patients at the time of initial diagnosis stage.

**Methodology:**

Herein, using RT-PCR based splice assays we have examined CD19 splicing patterns in 43 primary pediatric B-ALL patient samples spread across various subtypes, and investigated underlying mechanisms harboring aberrant splicing.

**Results:**

We observe that CD19 isoform lacking exon 5-6 is present in ~ 55% of pediatric patients at the initial diagnosis stage itself. Our in-silico analysis identified splicing regulator MBNL1 as a potential modulator of CD19 exon 5-6 splicing. Subsequent qRT-PCR analysis in patient samples revealed that MBNL1 is overexpressed in patient samples exhibiting exon 5-6 skipping. Furthermore, our functional studies demonstrate that loss of MBNL1 in B-ALL cell line induces exon 5-6 skipping, thereby confirming its mechanistic role in CD19 splicing regulation.

**Conclusions:**

Taken together, we for the first time report the existence of aberrantly spliced CD19 isoform lacking exon 5-6 in primary pediatric patients at the diagnosis stage. Our results suggest that this MBNL1 dysregulation contributes to this splicing event, potentially predisposing patients to resistance against CD19-directed immunotherapies.

**Supplementary Information:**

The online version contains supplementary material available at 10.1186/s40348-025-00207-y.

## Introduction

B-cell acute lymphoblastic leukemia (B-ALL) is an aggressive blood malignancy arising from the uncontrolled proliferation of immature B-cell lymphoblasts in the bone marrow leading to impaired hematopoiesis and immune dysfunction [1, 2]. Notably, B-ALL is the most common form of leukemia observed in children as around 80% of leukemia cases in children under the age of 15 are of B-ALL origin [3]. In last few decades, risk-adapted therapy has achieved cure rates exceeding 85% in pediatric patients and 40–60% in adults, however, certain subtypes such as KMT2A-rearranged, BCR::ABL1-positive (Ph +), BCR::ABL1-like (Ph-like), hypodiploid, iAMP21, and IKZF1-deleted B-ALL, remain associated with poor prognosis, treatment resistance, and higher relapse rates [4–10]. The use of chimeric antigen receptor (CAR)-T cells (CAR-T) therapy, a form of immunotherapy, as a treatment option against aggressive and refractory forms of B-ALL, has gained momentum in recent years [11, 12]. In CAR-T therapy for B-ALL, T-cells are genetically modified in a laboratory to express a CAR that specifically targets the CD19 antigen found on the surface of all leukemic B-cells. Subsequently, these modified T-cells are infused back into the patient's body intravenously, to effectively target and eliminate leukemic cells [13].

**Fig. 1:**
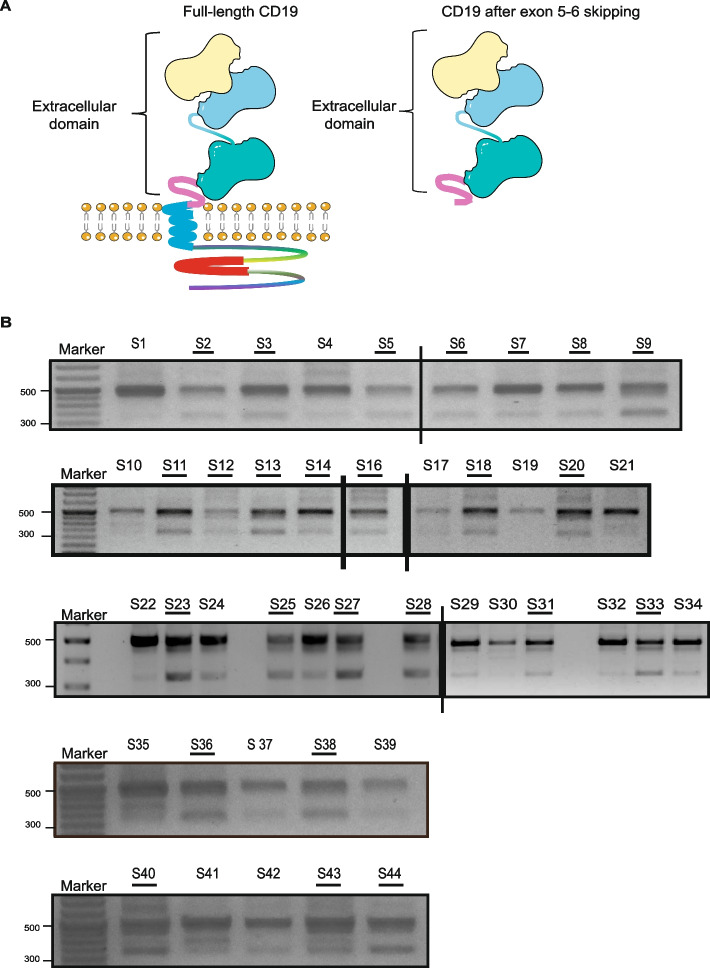
Full-length CD19 and truncated CD19 isoform. **a** The domain structure of the full-length CD19 protein includes extracellular, transmembrane, and cytosolic domains. Exons 5 and 6 are responsible for encoding the transmembrane and cytosolic domains, respectively. Skipping of these exons leads to the production of a truncated CD19 protein. **b** CD19 skipped exon 5–6 (SE5-6) isoform detection using semi-quantitative RT-PCR in primary pediatric B-ALL patients. Here 490 bp band represents full-length CD19 whereas 330 bp band represents CD19 SE5-6 isoform. Out of 43 patient samples of different subtypes, 25 samples had intense or faint SE5-6 bands along with full-length bands while 18 samples had only full length and no SE5-6 bands. Samples having intense or faint SE5-6 bands have been underlined in black. S1-S44 = primary pediatric B-ALL patient samples

Human CD19 gene contains 15 exons and is located on chromosome 16p11.2. It encodes for a 95 kDa transmembrane glycoprotein which contains extracellular Ig domains, a transmembrane domain, and a long cytoplasmic domain [14, 15]. CD19 is a pan B-cell antigen that is expressed in the earliest stages of B-cell development and is necessary for normal differentiation and maturation of B-cells [16]. Moreover, CD19 is a crucial marker for lineage assignment of blasts as per World Health Organization’s (WHO) guidelines (WHO blue book) and is present in almost all cases of B-ALL, making it the perfect target for CAR-Ts. Initially, CAR-T treatment demonstrated remarkable and encouraging outcomes, achieving a remission rate of approximately 90% in B-ALL patients [15]. However, over time, post CAR-T therapy, disease relapse was observed in ~ 30–50% of the patients [17, 18]. In most cases, relapse is attributed to the loss of the CD19 surface antigen, rendering the malignant B-cells unrecognizable to CAR-Ts [17, 19]. Various splicing events, such as exon or intron, skipping and retention have been suggested as processes causing damage to the CD19 epitope with many of these events occurring prior to any therapeutic interventions. Sotillo et al., studied relapsed CAR-T patient samples and proposed that skipping of CD19 exon 2 and exon 5–6 can lead to loss of canonical CD19 surface antigen, contributing towards CAR-T resistance [20]. Moreover, Fischer et al., analyzed primary B-ALL patient samples and reported that CD19 skipped exon 2 isoform can exist at diagnosis stage itself, however they didn’t observe skipping of exon 5–6 at the diagnosis stage [21]. Additionally, Asnani et al. analyzed CAR-T relapse samples and observed intron 2 retention in one of the relapse samples, suggesting that this splicing event may lead to either the production of a truncated CD19 protein or CD19 mRNA degradation via nonsense-mediated decay [22]. Similarily, Ziegler et al., reported that intron 2 retention is present in leukemic blasts of patients at diagnosis stage [23]. Currently, literature lacks studies on the existence of alternatively spliced isoforms with exon 5 and 6 exclusions in de novo primary pediatric B-ALL. While exon 2 encodes a significant fraction of CD19 epitope recognized by CAR-Ts, exons 5 and 6 of the CD19 protein encode for its transmembrane & cytosolic domains necessary for proper cell surface localization of the protein. Loss of these exons is predicted to result in an altered CD19 isoform which can escape detection by CAR-Ts (Fig. 1a) [21, 24].

In this study, we for the first time have analyzed the skipping of CD19 exon 5 and 6 (SE5-6) isoform in 43 cases of de novo pediatric B-ALL. Furthermore, using *in-silico* analysis we have identified the RNA-binding proteins (RBPs) that bind to the exon 5–6 loci and can potentially influence the skipping of exon 5–6. These RBPs are significantly upregulated in patients exhibiting exon 5–6 skipping.

## Materials and methods

### Patient sample collection and processing, FISH data analysis

This study comprised of 43 cases of de novo pediatric B-ALL patients. Ethical approvals were obtained (IEC Protocol No. IITH/IEC/2022/07/12) from the participating institutes. Bone marrow aspirate and peripheral blood samples were collected from patients with informed consent. Peripheral blood counts were recorded, and morphological assessments of blood & bone marrow aspirates were conducted to determine blast percentages. Patients with blast counts < 30% were excluded from this study. Samples underwent flow cytometric immunophenotyping using a BD FACSLyric instrument with antibodies targeting B, T, and myeloid lineage markers. The study included patients aged 1–12 years, while those under 1 year were excluded due to sample size constraints. Fluorescence in situ hybridization (FISH) was used to detect genetic abnormalities including BCR::ABL1, ETV6::RUNX1 fusions, KMT2A rearrangements, TCF3, ZNF384, CRLF2, CSF1R, PDGFRB, JAK2, ABL1 and ABL2. Samples negative for WHO defined abnormalities were classified as B-others.

### RNA isolation, cDNA preparation and PCR

RNA was extracted from the peripheral blood and/or bone marrow samples using QIAamp RNA Blood Mini Kit (Qiagen, Hilden, Germany) as per the manufacturer’s instructions. Following RNA isolation, cDNA preparation was done with 2ug of RNA using ProtoScript II Reverse Transcriptase (New England Biolabs) as per manufacturer’s instructions. To identify the SE5-6 transcripts in CD19, the following primer pair spanning exon 4 to exon 8 was used, forward: 5’ AAGGGGCCTAAGTCATTGCT 3’ and reverse: 5’ TGCTCGGGTTTCCATAAGAC 3’, as described by Sotillo et al. [20]. CD19 PCR of cDNA samples was done using 12.5ul of Amplitaq gold 360 mastermix (Thermo Scientific, Dreieich, Germany), 2uL of cDNA, 0.5uL of each forward and reverse primers, and 4.5uL of nuclease free water per reaction under following cycling conditions: Initial denaturation at 95^0^C for 10 min, followed by 35 cycles of denaturation 95^0^C for 30 secs, annealing 62^0^C for 30 secs, extension 72^0^C for 30 secs, and final extension of 72^0^C for 7 mints.

### Sequencing

Sanger sequencing was performed to confirm the nature of the bands obtained on gel electrophoresis. The bands were cut under UV transillumination. The band was later eluted from the gel using NucleoSpin Gel and PCR cleanup kit (Macherey–Nagel). The eluted product was cleaned using ExoSAP-IT™ (Thermo Scientific, Dreieich, Germany) and sequenced in Applied Biosystems 3500 genetic analyzer using BigDye™ Terminator v3.1 Cycle Sequencing Kit (Thermo Scientific, Dreieich, Germany). Germany). The data was analyzed and aligned with the CD19 transcript using Geneious Primer version 2023.2.1 software.

### *In-silico* analysis and qRT-PCR

To identify the RBPs interacting with CD19 exon 5–6 (chr16:28,935,221–28,936,411) locus we performed CD19 sequence analysis using RBPmap (https://rbpmap.technion.ac.il/) and oRNAment (https://rnabiology.ircm.qc.ca/oRNAment). RBPmap uses a weighted-rank technique to map the motifs present on RNA sequences and allows the user to choose their desired motifs [25]. oRNAment uses a search algorithm which provides a matrix similarity score between 0 an1 after scanning each transcript [26]. To shortlist RBPs, we had set the stringency level to highly stringent in the RBPmap and for oRNAment we selected RBPs with score > 0.1. Thereafter, qRT-PCR was used to assess the expression of shortlisted RBPs in the B-ALL patient samples. The primers used for qRT-PCR are listed in supplementary Table 1.

### Lentiviral transduction and shRNA driven knockdown

HEK293T cells were seeded in individual wells of a 6-well plate and incubated at 37 degrees. When HEK293T cells reached 50% confluency, they were co-transfected with the MBNL1 shRNA plasmids, control shRNA plasmid, the lentiviral envelope plasmid pMD2.G, and the packaging plasmid pSPAX2, using Effectene transfection reagent (Qiagen ID: 301425). For transfection, 500 ng of pMD2.G was used, while 1 µg of both pSPAX2 and the shRNA plasmids were used. The viral supernatant for infection in RS4;11 cells was harvested 48 h post transfection, by passing it through a 0.45uM filter. Subsequently, RS4;11 cells were infected with control shRNA lentivirus and MBNL1 shRNA lentivirus using 1 µg/ml of polybrene, and were selected using 0.75 ug/ml concentration of puromycin for 72 h. After puromycin removal, cells were allowed to recover and attain proper morphology, before using them for further experiments. shRNAs used for MBNL1 knockdown are listed in supplementary Table 1.

### Patient data analysis

RNA-Seq RPKM file containing gene expression data of 203 B-ALL patients was retrieved from the project: “Pediatric Acute Lymphoid Leukemia—Phase II (TARGET, 2018), deposited in online cancer repository cBioPortal (https://www.cbioportal.org/) [27]. Additionally, mRNA expression data for the shortlisted RBPs was also retrieved from cBioPortal using the same TARGET 2018 project.

### Statistical tests

Statistical tests (unpaired t-test) for qRT-PCR and expression analysis of shortlisted RBPs was done using GraphPad prism software. Correlation analysis of the shortlisted RBPs was also conducted using GraphPad Prism.

## Results

### Patient characteristics

Patient samples were collected at the time of initial diagnostic workup and all the patients included in the study exhibited CD19 expression. CD19 was moderately expressed in ~ 70% cases, around ~ 25% cases had dim to moderate CD19 expression, and less than 5% cases had low CD19 expression. The cohort predominantly comprised pediatric patients, with a mean age of 6.4 years (range: 1–12 years). Only two patients were older than 12 years, aged 17 and 20 years, respectively. Of total, 62% were males while 38% were females. Hemoglobin of the study population varied from 3 gm/dL to 13 gm/dL with 96% of the patients presenting with anemia (Hb < 11gm/dL) at diagnosis. Total leucocyte counts varied from 1 × 103 cells/uL to 232 × 103 cells/uL. 30% of the patients had leucopenia while 38% had leucocytosis. Except for one case, S37, all patients were thrombocytopenic. Blast percentages were higher in bone marrow samples as compared to peripheral blood. More than 50% blasts were present in peripheral blood and bone marrow, in 80% and 93.5% of the cases respectively. Patient characteristics are summarized in Table 1.

**Table 1:** Diagnostic lab findings and patient characteristics

Demographics				Diagnostic lab findings					
Sl. No	**RNA code**	**Age**	**Gender**	**Hb**	**WBC count × 103/uL**	**Platelets × 103/uL**	**Blast % in Peripheral blood**	**Blast % in bone marrow aspirate**	**Subtype**
1	S1	12	M	6.6	54.1	10	95	98	*BCR::ABL1*
2	S2	3	M	7.9	13.1	7	51	89	*BCR::ABL1*
3	S3	10	F	3	109.3	19	78	93	*BCR::ABL1*
4	S4	12	M	6.4	32.1	14	45	79	*TCF3* rearranged
5	S5	4	F	8.5	4	13	60	82	*TCF3* rearranged
6	S6	9	F	3.2	13.7	7	84	96	*ETV6::RUNX1*
7	S7	9	M	7.7	232.8	51	95	90	*ETV6::RUNX1*
8	S8	3	M	7.9	43.7	14	91	98	*ETV6::RUNX1*
9	S9	3	F	10.7	1.9	11	90	95	*ETV6::RUNX1*
10	S10	4	F	8.8	4.9	26	67	85	Hyperdiploidy
11	S11	2	F	5.8	120	28	40	95	Hyperdiploidy
12	S12	3.5	M	7.3	2.9	20	70	95	Hyperdiploidy
13	S13	7	M				87	85	Hyperdiploidy
14	S14	2	M	9.2	38.7	17	93	90	Hyperdiploidy
15	S15	4	M	4.7	126.8	27	75	85	Ph like ALL
16	S16	3	M	7.2	14.3	18	72	80	*TCF3* rearranged
17	S17	8	M	10.1	1.71	14	45	95	B-others
18	S18	3	M	6.4	66.2	25	96	97	B-others
19	S19	7	F	5.8	14	35	28	68	B-others
20	S20	6	M	8.3	1	8	86	90	B-others
21	S21	17	M	9.1	32.3	54	80	88	Ph like ALL
22	S22	20	F	6.7	11	18	65	98	*ETV6::RUNX1*
23	S23	3	F	7.4	17.8	27	61	81	Hyperdiploidy
24	S24	8	F	8.2	13	14	92	95	B-others
25	S25	4	M	5.2	8.4	10	68	85	B-others
26	S26	10	F	8.1	11.3	22	80	89	Hyperdiploidy
27	S27	3	M	6.8	82	15	86	95	B-others
28	S28	5	M	10.4	12.2	46	51	85	B-others
29	S29	3	F	9	8.2	68	NA	81	Hyderdiploidy
30	S30	5	M	9	3.05	75	70	21	Hyperdiploidy
31	S31	11	M	7.6	6	70	65	91	B-others
32	S32	5	F	6	31.9	10	93	95	Hyderdiploidy
33	S33	8	F	5.2	8.3	12	90	95	Hyderdiploidy
34	S34	8	M	5.3	135.2	7	92	95	B-others
35	S35	9	F	4.9	35.1	25	93	90	Hyderdiploidy
36	S36	3	M	12.4	33.7	19	82	96	Hyperdiplopidy
37	S37	9	M	13	5	249	2	32	Hyperdiplopidy
38	S38	5	M	8	4.9	17	59	95	Hyperdiplopidy
39	S39	5	M	9.2	4.9	47	72	92	Hyperdiplopidy
40	S40	4	M	8.1	34.5	11	92	95	Ph Like ALL
41	S41	3	M	10.7	27.9	21	95	74	B-others
42	S42	7	F	4.9	6.5	50	50	97	B-others
43	S43	9	M	10.7	8.64	99	40	76	B-others
44	S44	3	M	6.2	71.5	21	80	95	B-others

### CD19 splicing gels exhibit alternatively spliced isoform

We performed semiquantitative RT-PCR in the primary pediatric B-ALL patient samples spread across different subtypes, using primers spanning exons 4–8. Using these primers, the expected band size for full-length wild-type CD19 transcript is 490 bp size whereas a transcript with SE5-6 is expected to give a band size of 330 bp (exons 5 and 6 are 111 bp and 49 bp long, respectively). Strikingly, in our RT-PCR analysis, we observed that a significant percentage of patient samples demonstrated the presence of CD19 SE5-6 variant at the diagnosis stage itself, prior to any therapy (Fig. 1b). The samples exhibited variation in the intensity of full-length & SE5-6 transcripts, with most samples showing prominent full-length transcript compared to SE5-6 transcript (Fig. 1b). Next, we performed densitometric analysis to quantify CD19 full length and SE5-6 bands using ImageJ software. Based on densitometric analysis we set a threshold of 10% for SE5-6/full-length band intensity, above which a sample was considered to exhibit SE5-6 band while those below the threshold were considered as non-skipping samples (Supplementary Fig. 1a). We observed that out of our 43 patient samples, 25 samples exhibited the presence of SE5-6 band to varying degrees along with the full-length CD19 band, whereas 18 samples exhibited only full-length band and had no/background SE5-6 band. Furthermore, intense SE5-6 bands (SE5-6/full-length > 30%) were observed in around 25% samples such as: S9, S13, S23, S25, S27, S28, S33, S36, S38, S40 and S44. Notably, we observed that SE5-6 bands were present in primary patient samples irrespective of the subtype, i.e., the skipping was not subtype specific. Taken together our findings suggest that CD19 SE5-6 isoform responsible for the loss of canonical CD19 epitope, is already present in pediatric B-ALL patients across different subtypes, at the time of diagnosis itself, prior to any therapeutic intervention.

Furthermore, to confirm the nature of the obtained bands, the full-length & SE5-6 bands were gel eluted and sequenced using the Applied Biosystems 3500 genetic analyzer. The data was analyzed and aligned with CD19 transcript using Geneious Primer version 2023.2.1 software. The sequencing result validated the presence of SE5-6 isoform. The 490 bp band representing the full-length transcript mapped precisely on to the full length CD19 transcript with no gaps. The 330 bp size band representing the SE5-6 transcript mapped to exons 4, exon 7 and exon 8 of CD19 transcript with a gap of 160 bp (exon 5 is of 111 bp and exon 6 of 49 bp length – both skipped), thereby confirming the nature of the bands (Supplementary Fig. 1b-1c).

### MBNL1 regulates CD19 exon 5–6 splicing

**Fig. 2:**
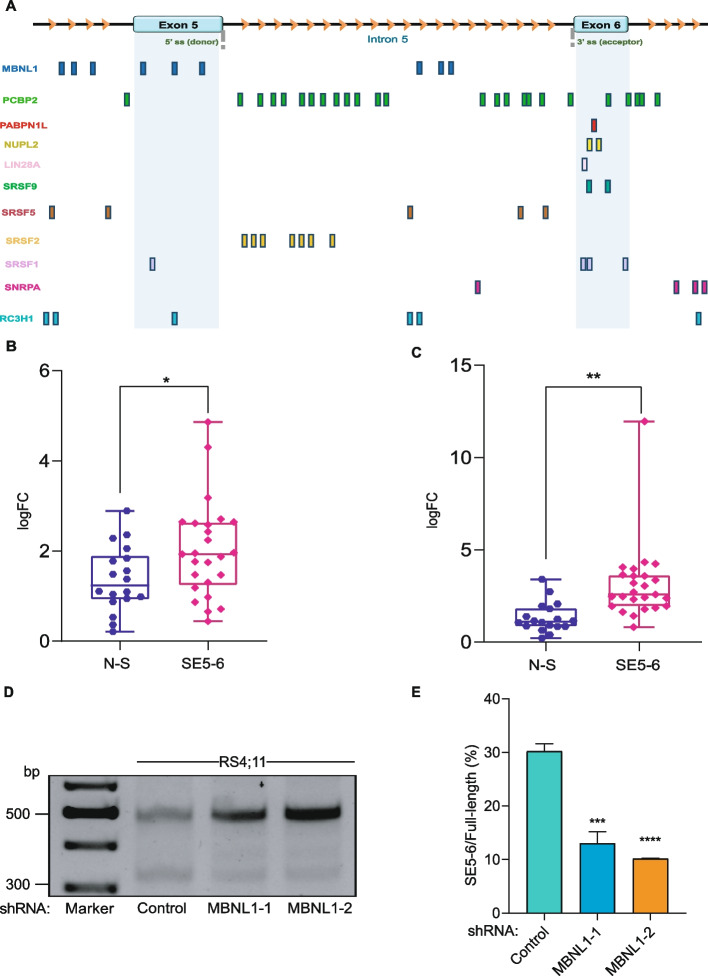
Identification of novel RBPs regulating CD19 exon 5–6 skipping. **a** Common RBPs obtained from public databases RBPmap and oRNAment along with their binding sites (from RBPmap-UCSC genome browser’s data) on CD19 exon 5–6 locus. The light blue regions indicates exon 5 and exon 6 regions respectively. **b** qRT-PCR showing that *MBNL1* is significantly upregulated (unpaired t-test, *p.value < 0.05) in B-ALL patient samples exhibiting skipped exon 5–6 (SE5-6) transcripts (*n* = 25) when compared to samples showing no skipping (N-S) bands (*n* = 18). **c** qRT-PCR showing that *RC3H1* is significantly upregulated (unpaired t-test, **p.value < 0.01) in B-ALL patient samples exhibiting skipped exon 5–6 (SE5-6) transcripts (n = 25) when compared to samples showing no skipping (N-S) bands (*n* = 18). Herein, S2, S3, S5, S6, S7, S8, S9, S11, S12, S13, S14, S16, S18, S20, S23, S25, S27, S28, S31, S33, S36, S38, S40, S43, S44 were taken as SE5-6 samples whereas S1, S4, S10, S17, S19, S21, S22, S24, S26, S29, S30, S32, S34, S35, S37, S39, S41, S42 samples were taken as non-skipping samples. **d** Agarose gel electrophoresis of semi-quantitative RT-PCR showing decrease in SE5-6 isoform intensity post MBNL1 knockdown. Here 490 bp band represents full-length CD19 whereas 330 bp band represents CD19 SE5-6 isoform. **e** Densitometric quantification of semi-quantitative RT-PCR showing decrease in CD19 exon 5–6 skipping post MBNL1 knockdown (unpaired t-test, ***p.value < 0.001, ****p.value < 0.0001)

RBPs are known to regulate exon inclusion and skipping by binding to exon splicing enhancer (ESE) sites in the pre-mRNA. Previous studies have reported that RBPs and related splice factors, regulate aberrant splicing events in CD19, for instance, Sotillo et al., reported that SRSF3 controls exon 2 inclusion in CD19 [20]. Additionally, multiple studies have reported that PTBP1 modulates retention of intron 2 in CD19 [23, 28]. To identify the RBPs that might interact with CD19 exon 5–6 locus and affect splicing, we performed RBP analysis by exploring publicly available RBPmap [29] and oRNAment [26] databases. To select putative RBPs, stringency level was set to highly stringent in RBPmap, and oRNAment score > 0.1 was considered. We shortlisted the common RBPs obtained from these databases using VENNY 2.1 (https://bioinfogp.cnb.csic.es/tools/venny/). BOLL, LIN28A, MBNL1, NUPL2, PABPN1L, PCBP2, RC3H1, SNRPA, SRSF1, SRSF2, SRSF5, SRSF9 were the 12 common RBPs identified using VENNY 2.1 (Supplementary Fig. 2). RBPs with more than 5 binding sites in the exon 5–6 locus (from RBPmap-UCSC genome browser’s data) and significantly linked with B-ALL pathophysiology were further shortlisted. MBNL1, PCBP2, RC3H1 and SRSF2 each with more than 5 binding sites were selected for further analysis due to their increased likelihood of binding (Fig. 2a). Further, to ascertain the correlation between expression levels of these four RBPs and B-ALL pathophysiology, we investigated “Pediatric Acute Lymphoid Leukemia—Phase II (TARGET, 2018)” data deposited in cBioPortal (https://www.cbioportal.org/). Our analysis revealed that expression levels of *MBNL1*, *PCBP2*, *RC3H1*, and *SRSF2* is significantly elevated in B-ALL patients exhibiting any alteration (such as mutations, truncations, copy number variations) in these RBPs compared to patients with no alterations (Supplementary Fig. 3a-3d). Subsequently, using qRT-PCR, we analyzed the expression of these four RBPs across our pediatric patient samples (non-skipping vs SE5-6 samples) and observed a strong correlation between the expression levels of *MBNL1* and *RC3H1* with exon skipping. *MBNL1* and *RC3H1* were significantly upregulated in patients exhibiting SE5-6 isoform compared to non-skipping samples, whereas *PCBP2* and *SRSF2* had no such change in their expression (Fig. 2b-c, Supplementary Fig. 4a-4b, respectively).

Further, we extracted and analyzed RNA-seq RPKM gene expression file of 203 B-ALL patients using the above mentioned TARGET-phase II pediatric ALL dataset and found that *RC3H1* and *MBNL1* are significantly correlated with each other (Supplementary Fig. 4c). This correlation of *RC3H1* and *MBNL1* is in line with our findings which shows upregulation of both the RBPs in samples exhibiting SE5-6 isoform.

Thereafter, from our two potential biomarkers, MBNL1 and RC3H1, we took MBNL1 for further analysis, given the role of MBNL1 as a canonical splice factor involved in oncogenic aberrant splicing [30]. RC3H1 on the other hand has E3 ligase property and it binds mostly to mRNAs induced in response to DNA damage [31], hence it wasn't considered for our further experiments. Thereupon, we used RS4;11 cells to generate cell lines that stably express shRNAs targeting MBNL1, to assess the effects of MBNL1 depletion on SE5-6 isoform. The knockdown of MBNL1 was validated using qRT-PCR, which showed ~ 50% reduction in MBNL1 expression for both the shRNAs targeting MBNL1 (Supplementary Fig. 4 d). Intriguingly, we found that depletion of MBNL1 significantly affects exon 5–6 skipping as shRNAs targeting MBNL1 exhibited reduced intensity of SE5-6 isoform compared to control shRNA (Fig. 2d-2e). Both, shRNA MBNL1-1 and shRNA MBNL1-2 had more than 50% reduction in SE5-6 isoform intensity compared to control shRNA. This result corroborates that enhanced levels of MBNL1 is associated with a CAR-T relapse related phenotype, which can damage the CD19 epitope on the B-cell surface. Hence, these findings suggest that MBNL1 is a potential biomarker whose depletion prevents skipping of CD19 exon 5–6.

Taken together, our results imply that MBNL1 and RC3H1 are potential regulators of exon 5–6 skipping in B-ALL patients, and attractive targets to enhance CAR-T therapy efficacy. However, more in-depth analysis is required to substantiate it.

## Discussion and conclusion

Alternative splicing plays a crucial role in regulating proteome diversity. Alterations in the splicing process results in the production of aberrantly spliced isoforms which are known to drive disease progression and drug resistance [32, 33]. Tumor cells exploit the presence of these irregularly spliced isoforms as a strategy to evade anti-cancer treatments [34, 35]. CD19 is a signaling antigen which plays a significant role in B-cell development, making it an attractive target for CAR-T therapy [36]. CAR-T therapy has shown immense promise in treating B-ALL patients but a significant percentage of patients exhibit aggressive relapse of B-ALL post CAR-T treatment [37]. Aberrant splicing of CD19 which leads to a truncated CD19 protein has been identified as a factor contributing to resistance against CAR-T therapy [33]. The primary objective of our study was to conduct a comprehensive examination of CD19 splicing in primary pediatric patient samples and get insights into the presence of altered CD19 isoforms in patients prior to any therapy. Our results demonstrate that SE5-6 variant of CD19 is present in primary pediatric B-ALL patients irrespective of the subtype. Exons 5 and 6 encode the transmembrane and cytosolic domains of CD19, respectively., Therefore, an isoform lacking these exons would not present the normal CAR-T epitope, enabling it to avoid detection by CAR-Ts. In our study, we observed that skipping of exon 5–6 in CD19 results in generation of a pre-mature stop codon leading to absence of transmembrane & cytosolic domains in CD19. Additionally, it has been shown that phosphorylation of tyrosine residues (Y482 and Y513) within the cytosolic domains of CD19 regulates intracellular signaling cascades essential for B-cells maturation and survival, highlighting the importance of cytosolic domain in B-cell development [38].

Mechanistically, RBPs control alternative splicing program by interacting with cis-acting elements within the pre-mRNA, thereby regulating exon skipping and/or inclusion. Consequently, any alteration in RBP activity can potentially lead to loss of target CD19 epitope, as suggested by earlier studies as well [22, 23, 28]. To investigate whether dysregulation in RBP function is linked with CAR-T-relapse associated CD19 isoforms, we performed both *in-silico* and *in-vitro* analysis. Our findings suggest that two RBPs MBNL1 & RC3H1, are significantly overexpressed in primary B-ALL patient samples exhibiting SE5-6 isoform, implying that ectopic expression of RBPs is indeed correlated with therapy-resistant isoforms. Analysis of TARGET-phase II pediatric ALL dataset deposited on cBioPortal revealed that RC3H1 and MBNL1 are moderately correlated with each other, which aligns with our analysis of patient samples. Given the involvement of MBNL1 in canonical splicing, and E3 ligase like function of RC3H1, we prioritized MBNL1 for our cell lines study. Subsequent knockdown of MBNL1 in RS;411 cell line revealed that MBNL1 depletion in turn diminishes SE5-6 isoform, which further substantiates the patient samples analysis where MBNL1 upregulation is associated with enhanced exon 5–6 skipping. Akin to this, several previous studies have also emphasized the role of MBNL1 in modulating B-ALL pathogenesis. MBNL1 knockdown promotes retention of CD19 intron 2 [28] and suppresses levels of B-ALL markers genes has been observed previously [39]. In addition, MBNL1 regulates aberrant splicing of genes involved in KMT2A rearranged oncogenesis has also been established [30]. Interestingly, a recent retrospective study reported that patients with KMT2A subtype have very poor overall survival post CAR-T therapy relapse [40]. These findings imply that MBNL1 dysregulation may be associated with poor response towards CAR-T therapy, directly or indirectly. Additionally, RC3H1 has also been found to hinder anti-tumor immunity, and mutations in RC3H1 have been linked to immune system dysregulation [41, 42]. However, one of the limitation of our study is that we were not able to perform DNA-sequencing on our patient samples to correlate mutations in CD19 with the presence of SE5-6 isoform. Our future studies will try to address this bottleneck. Additionally, further research is needed to elucidate whether dysregulation of splice factors alone or a broader set of genes, is responsible for driving CAR-T-relapse associated CD19 isoforms. To summarize, our *in-silico* and *in-vitro* data suggests that MBNL1 and RC3H1 may serve as key biomarkers for CAR-T resistance. These findings warrant the need for in-depth studies to robustly substantiate their role in CD19 epitope loss and to develop targeted therapeutics to prevent aberrant splicing.

Hence, to conclude, our study investigates the presence of aberrantly spliced CD19 isoform responsible for CAR-T resistance, in a large cohort of primary pediatric B-ALL patients, and sheds light on the RBPs regulating this aberrant splicing. We hypothesize that the presence of this isoform at diagnosis, may predispose patients to CAR-T therapy failure, as the isoform could become more dominant under therapeutic pressure. In future, the presence of this isoform in the diagnosis stage itself, can be used as a potential screening criterion to provide more personalized or combination therapy to the patients. In addition, checking the expression levels of RBPs at screening stage can also help in identifying the patients predisposed to CAR-T therapy failure.

## Supplementary Information

Supplementary Material 1: Figure S1. (a) ImageJ quantification of CD19 full-length and skipped exon 5-6 (SE5-6) bands obtained on the gel using semi-quantitative RT-PCR. Samples having SE5-6/full-length band intensity >10% threshold were considered as samples with legitimate SE5-6 band whereas samples having SE5-6/full-length band intensity below this threshold were considered as non-skipping samples. S1-S44 are the primary pediatric patient samples, whereas CR1 and CR2 are the control samples (b) Chromatogram of gel extracted CD19 full-length (490bp) and CD19 SE5-6 (330bp) obtained post sequencing, confirms the skipping of exon 5-6. (c) Multiple sequence alignment of sequencing result of CD19 full-length and SE5-6 isoform bands extracted from the gel also confirms the skipping of exon 5-6. CD19FL represents our obtained 490bp transcript mapping completely with the CD19 transcript amplified by primers spanning exon 4-8, without any gaps. CD19SE5-6 represents our obtained 330bp transcript mapping to exons 4, exon 7 and exon 8 of CD19 transcript where exon 5 (111bp) and exon 6 (49 bp) both are skipped.

Supplementary Material 2: Figure S2. Binding sites of shortlisted common commonRBPs downloaded from UCSC genome browser.

Supplementary Material 3: Figure S3. cBioportal’s “Pediatric Acute Lymphoid Leukemia - Phase II (TARGET, 2018)” dataset analysis. (a) *MBNL1* is significantly upregulated in B-ALL patients having any *MBNL1* alteration compared to unaltered patients (unpaired t-test, ****p.value < 0.0001). (b) *PCBP2* is significantly upregulated in B-ALL patients having any *PCBP2* alteration compared to unaltered patients (unpaired t-test, ****p.value < 0.0001). (c) *RC3H1* is significantly upregulated in B-ALL patients having any *RC3H1* alteration compared to unaltered patients (unpaired t-test, ****p.value < 0.0001). (d) *SRSF2* is significantly upregulated in B-ALL patients having any *SRSF2* alteration compared to unaltered patients (unpaired t-test, ****p.value < 0.0001). Here, altered group represent patients who had mutations, truncations and/or copy number variations whereas unaltered group represents patients who had none of these.

Supplementary Material 4: Figure S4. (a) qRT-PCR results indicate that there is no significant difference (unpaired t-test, *p*.value >0.05) in *SRSF2* expression levels in patients exhibiting CD19 SE5-6 transcript when compared to non-skipping samples. (b) qRT-PCR results indicate that there is no significant difference (unpaired t-test, *p*.value >0.05) in *PCBP2* expression levels in patients exhibiting CD19 SE5-6 transcript when compared to non-skipping samples. Herein, S2, S3, S5, S6, S7, S8, S9, S11, S12, S13, S14, S16, S18, S20, S23, S25, S27, S28, S31, S33, S36, S38, S40, S43, S44 were taken as SE5-6 samples whereas S1, S4, S10, S17, S19, S21, S22, S24, S26, S29, S30, S32, S34, S35, S37, S39, S41, S42 samples were taken as non-skipping samples. (c) Correlation analysis of RNA-Seq RPKM gene expression data extracted from “Pediatric Acute Lymphoid Leukemia - Phase II (TARGET, 2018)” deposited in cBioPortal, performed using GraphPad prism, shows that expression levels of *RC3H1* and *MBNL1* are moderately correlated with each other (Pearson coefficient (r) = 0.47, *p*.value < 0.01). (d) qRT-PCR quantification of MBNL1 knockdown in RS4;11 cell line (unpaired t-test, *p.value < 0.05).

## Data Availability

No datasets were generated or analysed during the current study.
