# Supplementary figures and images for: Widespread prevalence of CD19 exon 5–6 skipping in primary pediatric B-Cell acute lymphoblastic leukemia patients
Source: Mol Cell Pediatr. 2025 Nov 17;12:20. doi: 10.1186/s40348-025-00207-y (PMC12623574; doi:10.1186/s40348-025-00207-y)

Supplementary figure 1:

a

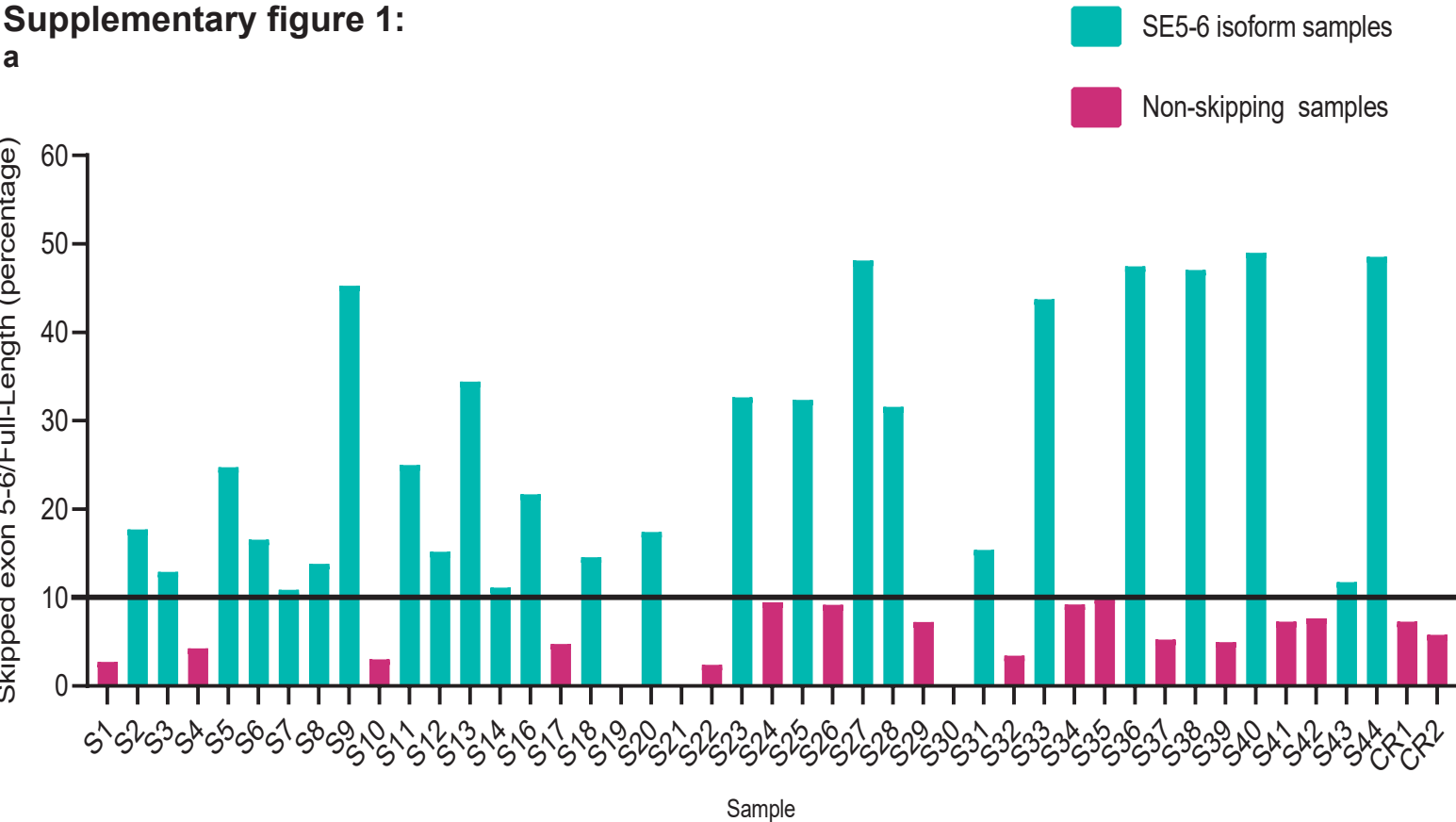

Supplement: Supplementary file 1 — Supplementary Material 1: Figure S1. (a) ImageJ quantification of CD19 full-length and skipped exon 5-6 (SE5-6) bands obtained on the gel using semi-quantitative RT-PCR. Samples having SE5-6/full-length band intensity >10% threshold were considered as samples with legitimate SE5-6 band whereas samples having SE5-6/full-length band intensity below this threshold were considered as non-skipping samples. S1-S44 are the primary pediatric patient samples, whereas CR1 and CR2 are the control samples (b) Chromatogram of gel extracted CD19 full-length (490bp) and CD19 SE5-6 (330bp) obtained post sequencing, confirms the skipping of exon 5-6. (c) Multiple sequence alignment of sequencing result of CD19 full-length and SE5-6 isoform bands extracted from the gel also confirms the skipping of exon 5-6. CD19FL represents our obtained 490bp transcript mapping completely with the CD19 transcript amplified by primers spanning exon 4-8, without any gaps. CD19SE5-6 represents our obtained 330bp transcript mapping to exons 4, exon 7 and exon 8 of CD19 transcript where exon 5 (111bp) and exon 6 (49 bp) both are skipped. [file 40348_2025_207_MOESM1_ESM.pdf]

Supplementary figure 2:

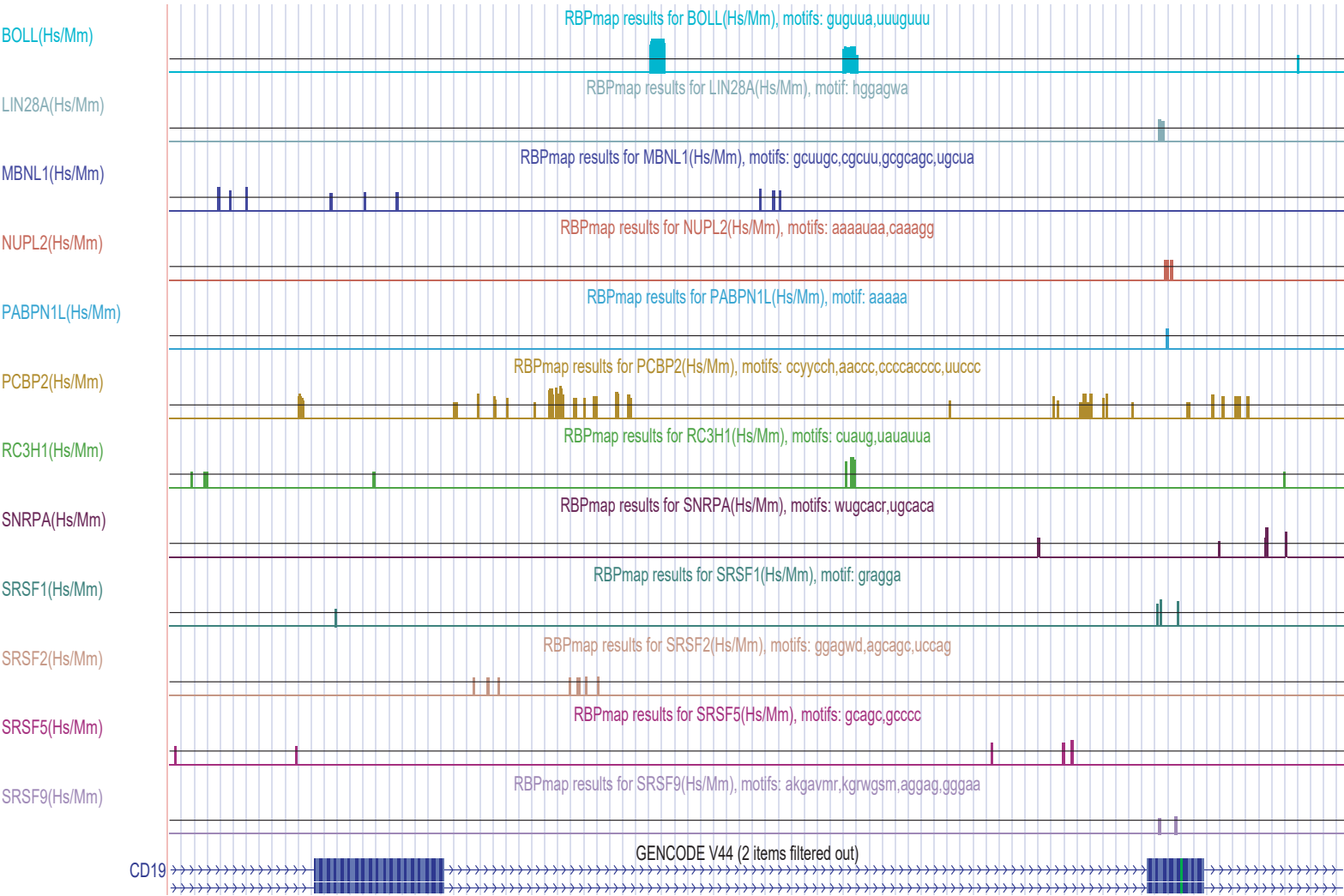

Supplement: Supplementary file 2 — Supplementary Material 2: Figure S2. Binding sites of shortlisted common commonRBPs downloaded from UCSC genome browser. [file 40348_2025_207_MOESM2_ESM.pdf]

Supplementary figure 3:

a

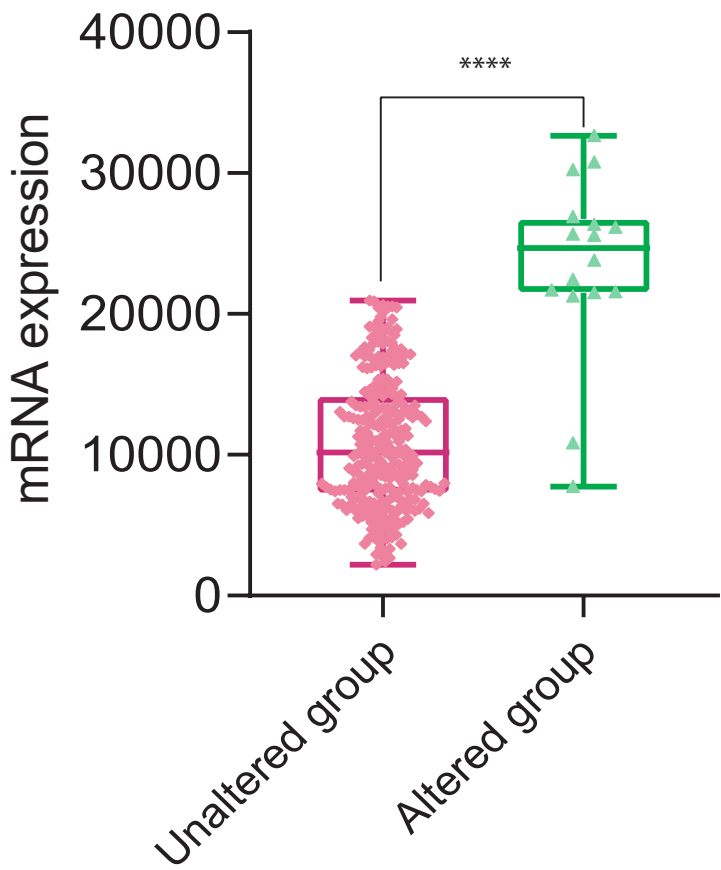

b

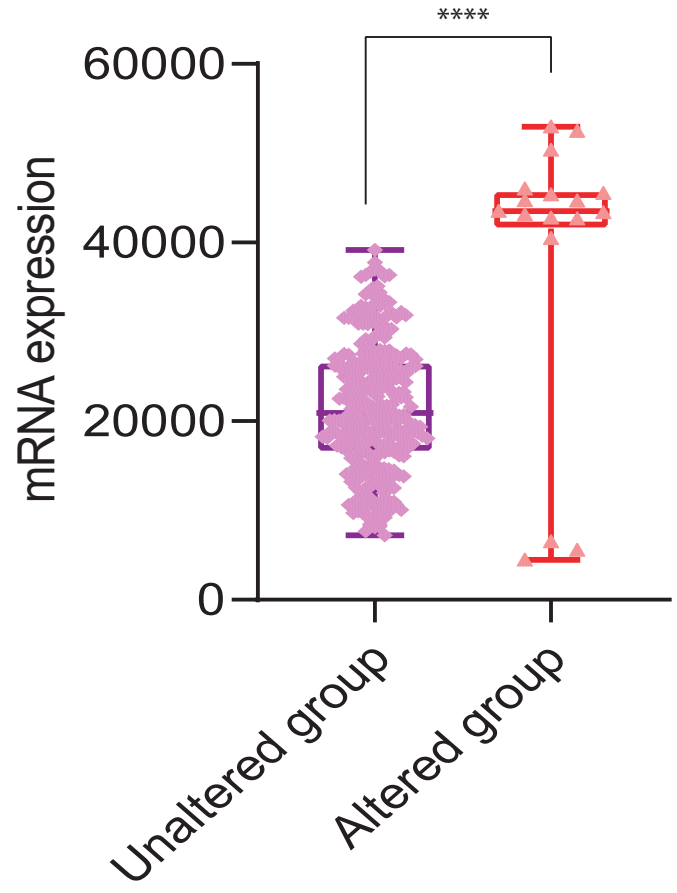

c

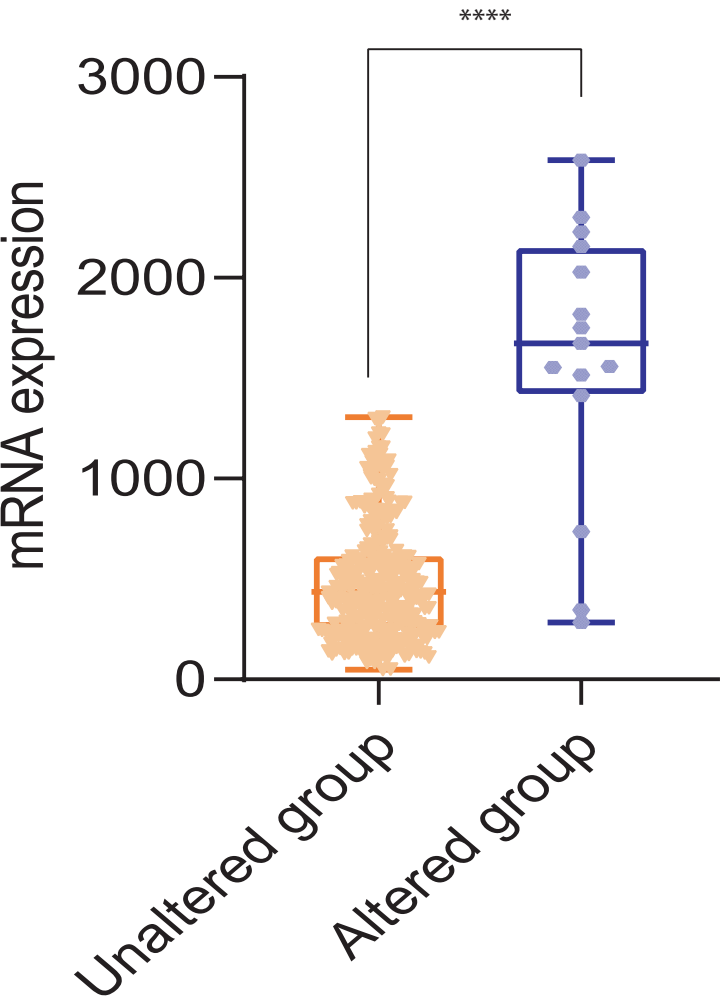

d

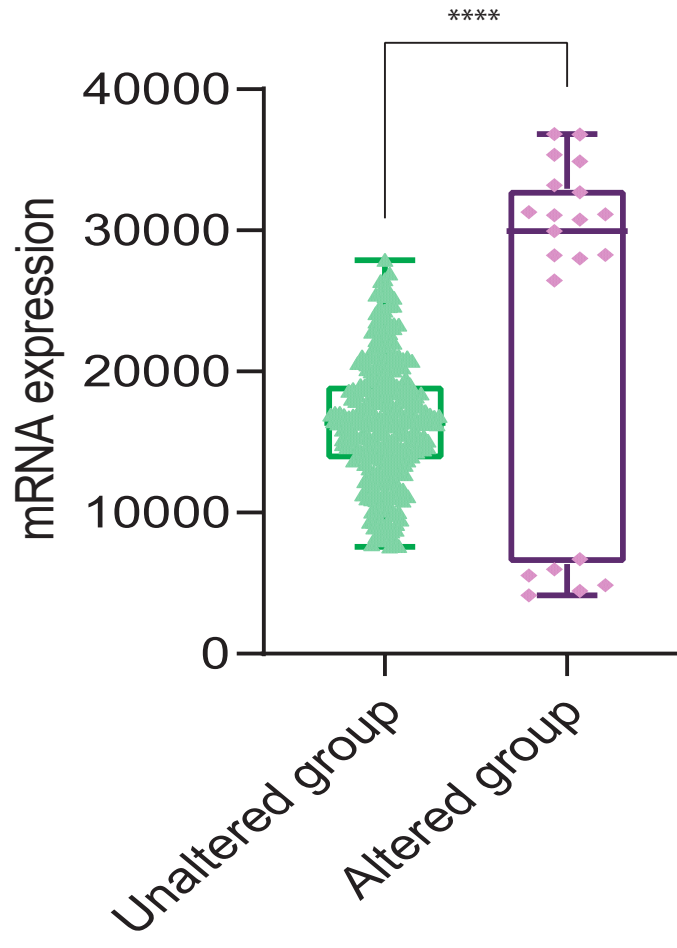

Supplement: Supplementary file 3 — Supplementary Material 3: Figure S3. cBioportal’s “Pediatric Acute Lymphoid Leukemia - Phase II (TARGET, 2018)” dataset analysis. (a) MBNL1 is significantly upregulated in B-ALL patients having any MBNL1 alteration compared to unaltered patients (unpaired t-test, ****p.value < 0.0001). (b) PCBP2 is significantly upregulated in B-ALL patients having any PCBP2 alteration compared to unaltered patients (unpaired t-test, ****p.value < 0.0001). (c) RC3H1 is significantly upregulated in B-ALL patients having any RC3H1 alteration compared to unaltered patients (unpaired t-test, ****p.value < 0.0001). (d) SRSF2 is significantly upregulated in B-ALL patients having any SRSF2 alteration compared to unaltered patients (unpaired t-test, ****p.value < 0.0001). Here, altered group represent patients who had mutations, truncations and/or copy number variations whereas unaltered group represents patients who had none of these. [file 40348_2025_207_MOESM3_ESM.pdf]

Supplementary figure 4:

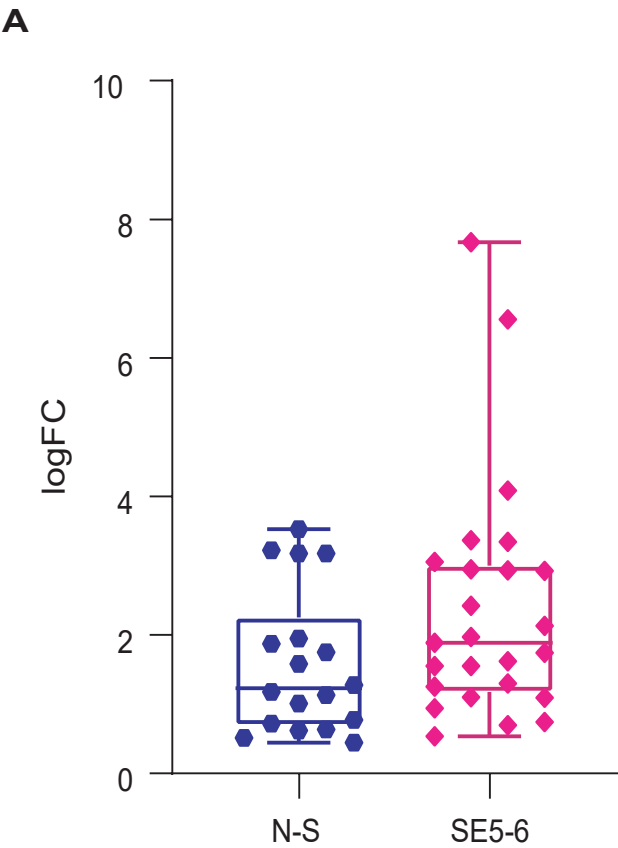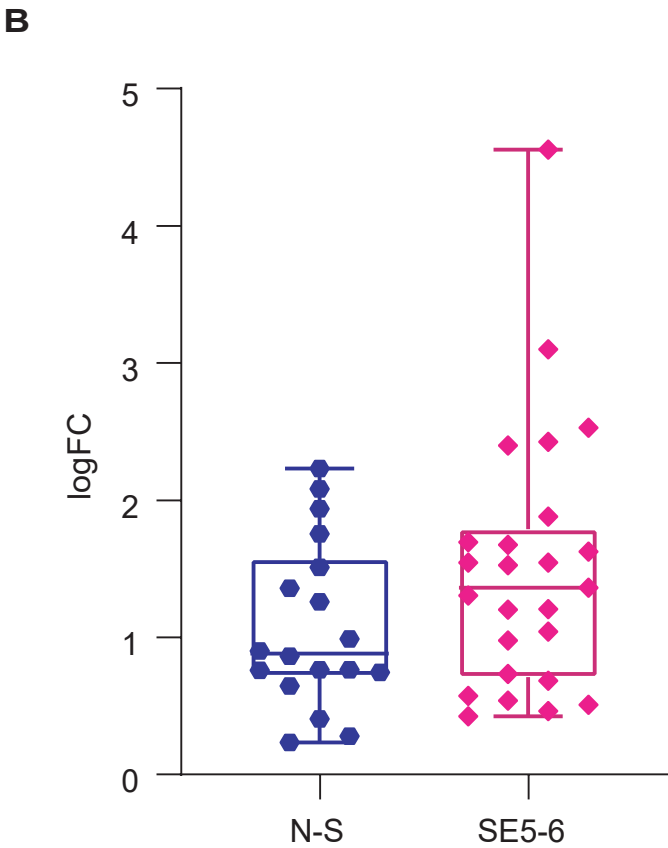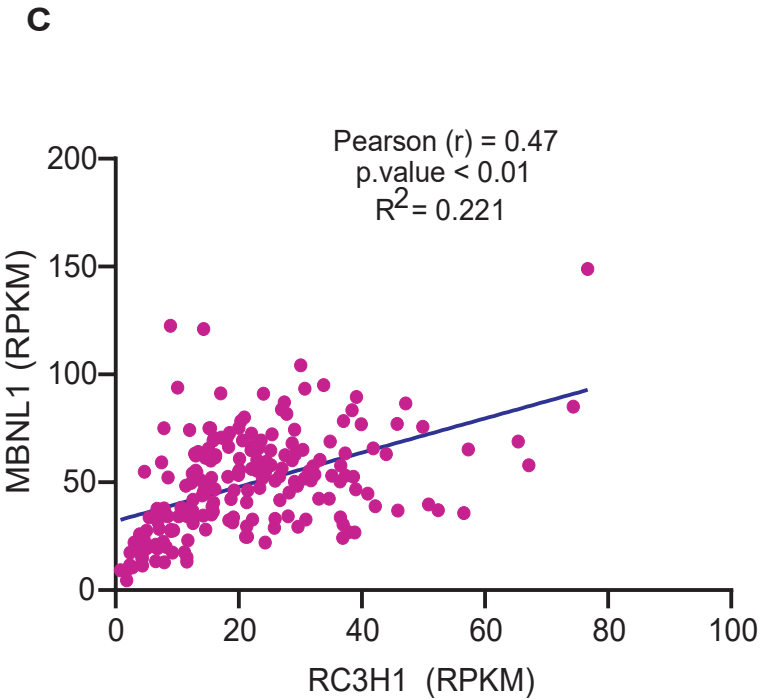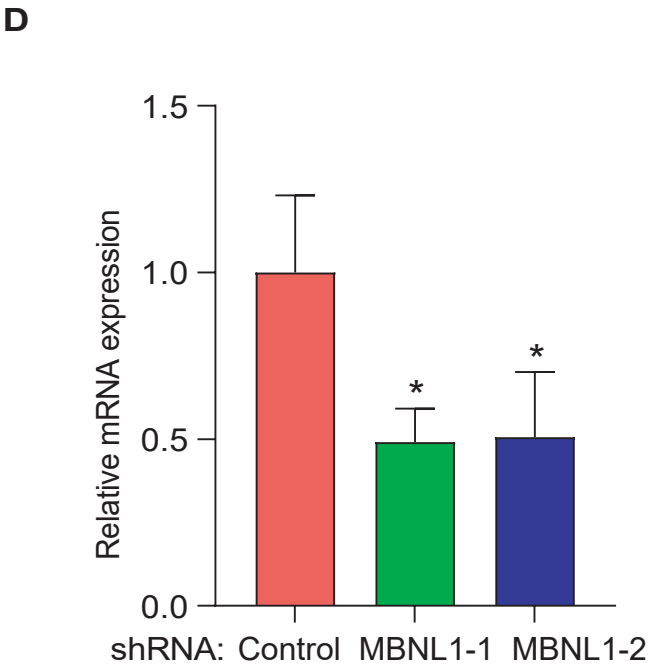

Supplement: Supplementary file 4 — Supplementary Material 4: Figure S4. (a) qRT-PCR results indicate that there is no significant difference (unpaired t-test, p.value >0.05) in SRSF2 expression levels in patients exhibiting CD19 SE5-6 transcript when compared to non-skipping samples. (b) qRT-PCR results indicate that there is no significant difference (unpaired t-test, p.value >0.05) in PCBP2 expression levels in patients exhibiting CD19 SE5-6 transcript when compared to non-skipping samples. Herein, S2, S3, S5, S6, S7, S8, S9, S11, S12, S13, S14, S16, S18, S20, S23, S25, S27, S28, S31, S33, S36, S38, S40, S43, S44 were taken as SE5-6 samples whereas S1, S4, S10, S17, S19, S21, S22, S24, S26, S29, S30, S32, S34, S35, S37, S39, S41, S42 samples were taken as non-skipping samples. (c) Correlation analysis of RNA-Seq RPKM gene expression data extracted from “Pediatric Acute Lymphoid Leukemia - Phase II (TARGET, 2018)” deposited in cBioPortal, performed using GraphPad prism, shows that expression levels of RC3H1 and MBNL1 are moderately correlated with each other (Pearson coefficient (r) = 0.47, p.value < 0.01). (d) qRT-PCR quantification of MBNL1 knockdown in RS4;11 cell line (unpaired t-test, *p.value < 0.05). [file 40348_2025_207_MOESM4_ESM.pdf]
